# Intrahepatic cholangiocarcinoma with extensive intraductal extension of high-grade biliary intraepithelial neoplasia: a case report

**DOI:** 10.1186/s40792-023-01748-y

**Published:** 2023-09-18

**Authors:** Haruna Kubota, Yasushi Hashimoto, Kazuhiro Toyota, Raita Yano, Hironori Kobayashi, Yujiro Yokoyama, Yoshihiro Sakashita, Kiyomi Taniyama, Katsunari Miyamoto, Yoshiaki Murakami

**Affiliations:** 1 Department of Surgery, Hiroshima Memorial Hospital, Honkawa-cho1-4-3, Naka-ku, Hiroshima, 730-0802 Japan; 2 Department of Pathology, Hiroshima Memorial Hospital, Hiroshima, Japan

**Keywords:** Intrahepatic cholangiocarcinoma, biliary intraepithelial neoplasia (BilIN), Intraductal extension, Precursor

## Abstract

**Background:**

Intrahepatic cholangiocarcinoma (ICC) is frequently associated with precursor lesions, and biliary intraepithelial neoplasia (BilIN) may play a significant role in the development of ICC. However, the exact sequence and progression of these lesions remain to be elucidated. We report a rare case of ICC that exhibited extensive longitudinal intraductal extension of high-grade BilIN in the posterior bile ducts and involved the hepatic hilum and the peripheral hepatic parenchyma.

**Case presentation:**

A 70-year-old female presented with anorexia. Computed tomography (CT) revealed a 15 mm enhancing intrahepatic tumor extending to the right intrahepatic secondary confluence. This was associated with a 7 mm diameter cystic dilatation of the segment 6 bile duct (B6). Endoscopic retrograde cholangiopancreatography (ERCP) revealed stenosis at the bifurcation of the posterior bile duct branch. Bile cytology confirmed the diagnosis of adenocarcinoma cells. Therefore, the patient was diagnosed with an ICC involving the right glissonean pedicle and underwent a right hepatectomy and lymph node dissection. Histologic examination revealed the tumor consisted of moderately differentiated adenocarcinoma. In connection with this lesion, diffuse intraductal atypical epithelial cells, which were diagnosed as high-grade BilIN, was observed not only in the dilated B6 but in the entire posterior bile ducts, which measured approximately 120 mm in diameter. Furthermore, two distinct foci of adenocarcinomas were identified in the peripheral hepatic parenchyma. A lymph node metastasis was also present. The pathological diagnosis was ICC pT4N1M0 stage IVA. The patient underwent adjuvant chemotherapy and has shown no recurrence 5 years after surgery. Imaging modalities were unable to accurately assess the extent of the intraductal neoplastic lesions due to their low papillary or sessile intraductal tubular growth. No risk factors for BilIN development, which has the potential to predispose to cholangiocarcinoma, were identified in the present case.

**Conclusions:**

We present a case of ICC involving the right hepatic hilum, accompanied by extensive longitudinal extensions of high-grade BilIN and multifocal microscopic invasions in peripheral hepatic parenchyma. Notably, the intraductal lesions involved the entire posterior intrahepatic bile ducts. The presence of biliary neoplasia with extensive intraductal extension, in conjunction with ICC, should be considered as a variant of BilIN.

## Background

Intrahepatic cholangiocarcinoma (ICC) is a neoplasm that originates from the endothelial cells of segmental or proximal branches of the bile duct, accounting for 5–20% of all primary liver malignancies [1–3]. Similar to the sequence model of pancreatic carcinoma, cholangiocarcinoma is presumed to follow a stepwise process of carcinogenesis involving precursor lesions such as biliary intraepithelial neoplasia (BilIN) and intraductal papillary neoplasm of the bile duct (IPNB) [4–6]. Pathogenesis of BilIN involves prolonged inflammation followed by sequential accumulation of genetic and epigenetic alterations and malignant transformation of the biliary epithelium. Most data are collected in association with other conditions, such as hepatolithiasis, primary sclerosing cholangitis (PSC), cirrhosis, etc. [6–9]. Multi-step carcinogenesis has been suggested in ICCs arising from BilIN and IPNB [4, 8]. However, little is known about the phenotypic or genetic changes during their carcinogenetic pathways, because only a few studies regarding both BilIN and IPNB have been conducted. Morphologically, ICC can be categorized into three main types based on the growth patterns: mass forming (MF), periductal infiltrative (PI), or intraductal growth (IG) [1, 10]. BilIN can only be identified under a microscope and cannot be classified into any of the macroscopic types. We present a rare case of ICC at right hepatic hilum that was associated with extensive longitudinal extension of high-grade BilIN lesions and multifocal distinct microscopic invasions in peripheral hepatic parenchyma.

## Case presentation

**Fig. 1:**
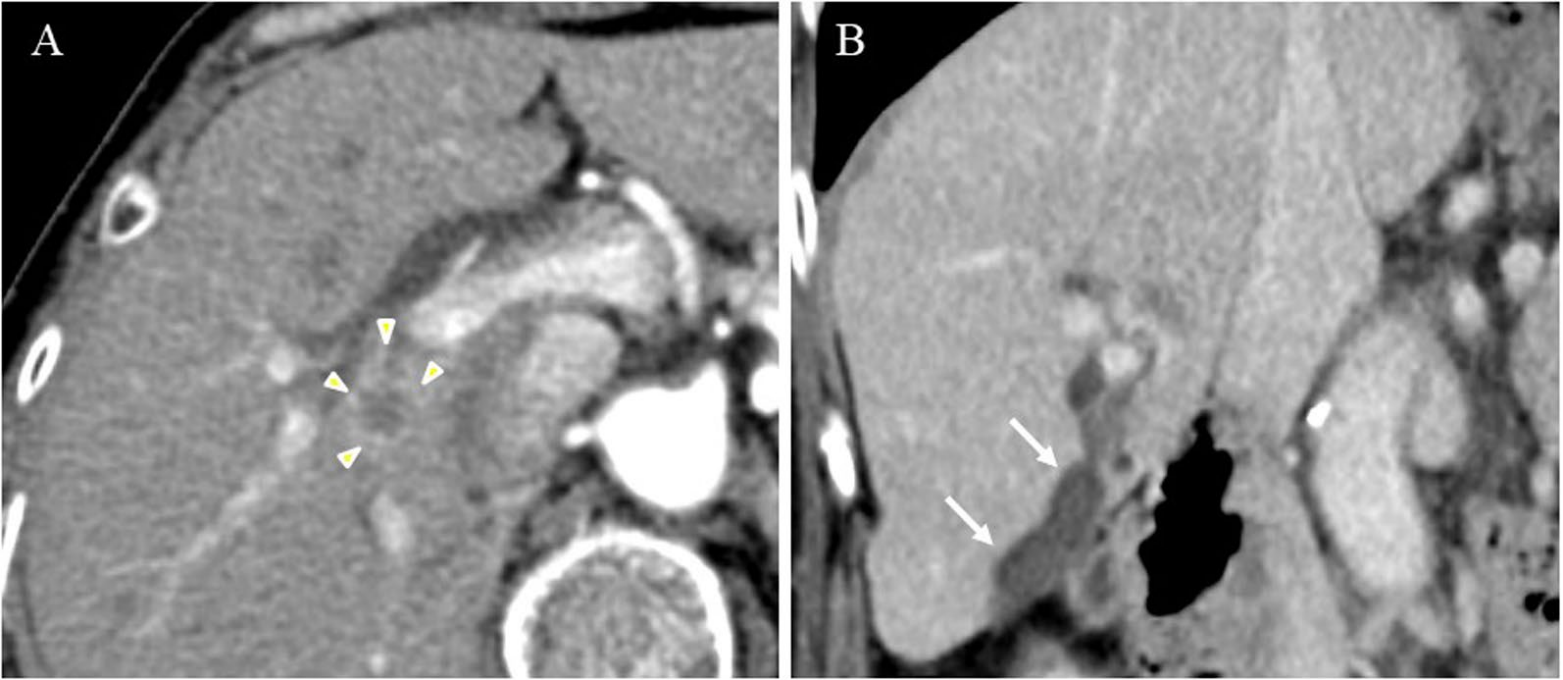
Preoperative contrast-enhanced CT findings. **A** In the axial view of the arterial phase, a 15 mm irregular circumferential ring-enhancing mass lesion was observed on the dorsal side of the right hepatic hilum (arrowheads). **B** In the coronal view of the venous phase, a cystic dilatation of the bile duct measuring 7 mm in diameter was observed in segment 6 bile ducts (B6) (arrows)

**Fig. 2:**
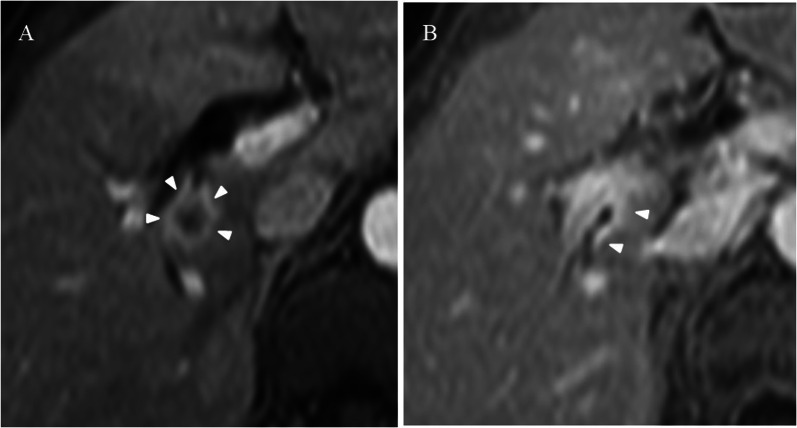
MRI findings. **A** In the axial section of contrast-enhanced T1-weighted images in the early phase, an irregular circumferential ring-enhancing mass was observed at the right hepatic hilum (arrowheads). **B** In the axial section at a level of the posterior glissonean confluence, marked wall thickening with enhancement was noted at the bifurcation of the posterior glissonean pedicle (arrowheads)

**Fig. 3:**
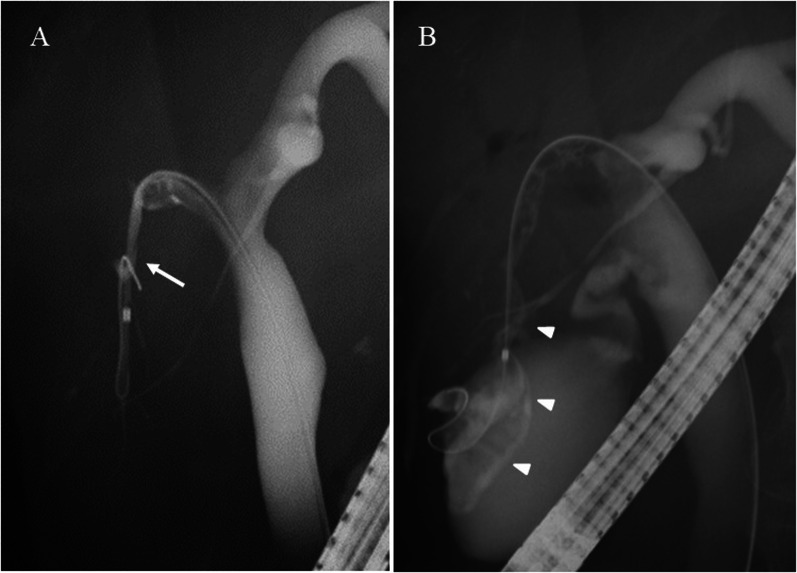
Endoscopic retrograde cholangiography (ERC) image. **A** Cholangiography showed a severe stenosis at the bifurcation of the segment 6 (B6) and 7 (B7) bile duct (arrow). **B** After cannulating the catheter, cystic dilatation of the segment 6 bile ducts (B6) was observed (arrowheads)

**Fig. 4:**
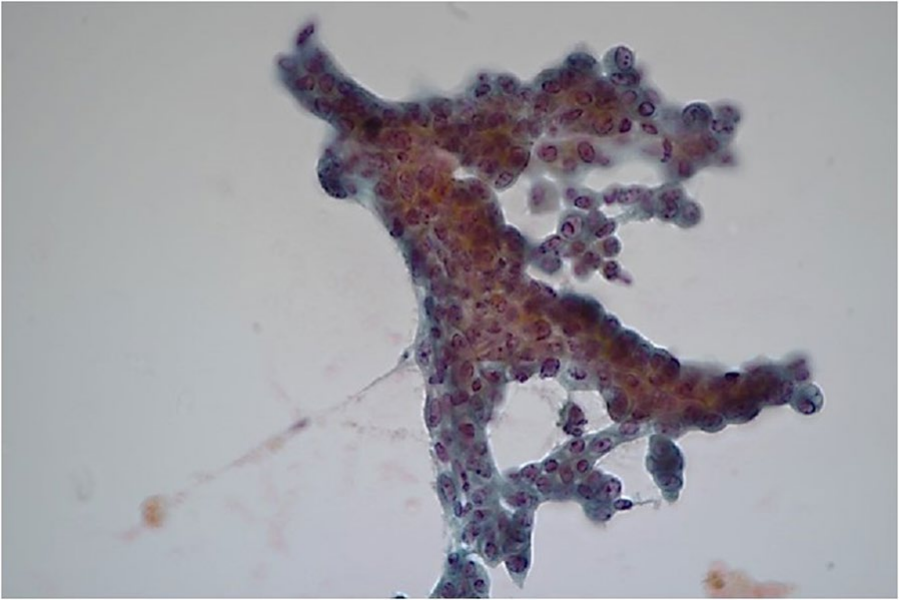
Bile cytology. The cytology examination revealed clusters of epithelial cells with features, such as anisokaryosis and hyperchromatic nuclei with irregular shape. This finding definitively confirmed the presence of adenocarcinoma cells

**Fig. 5:**
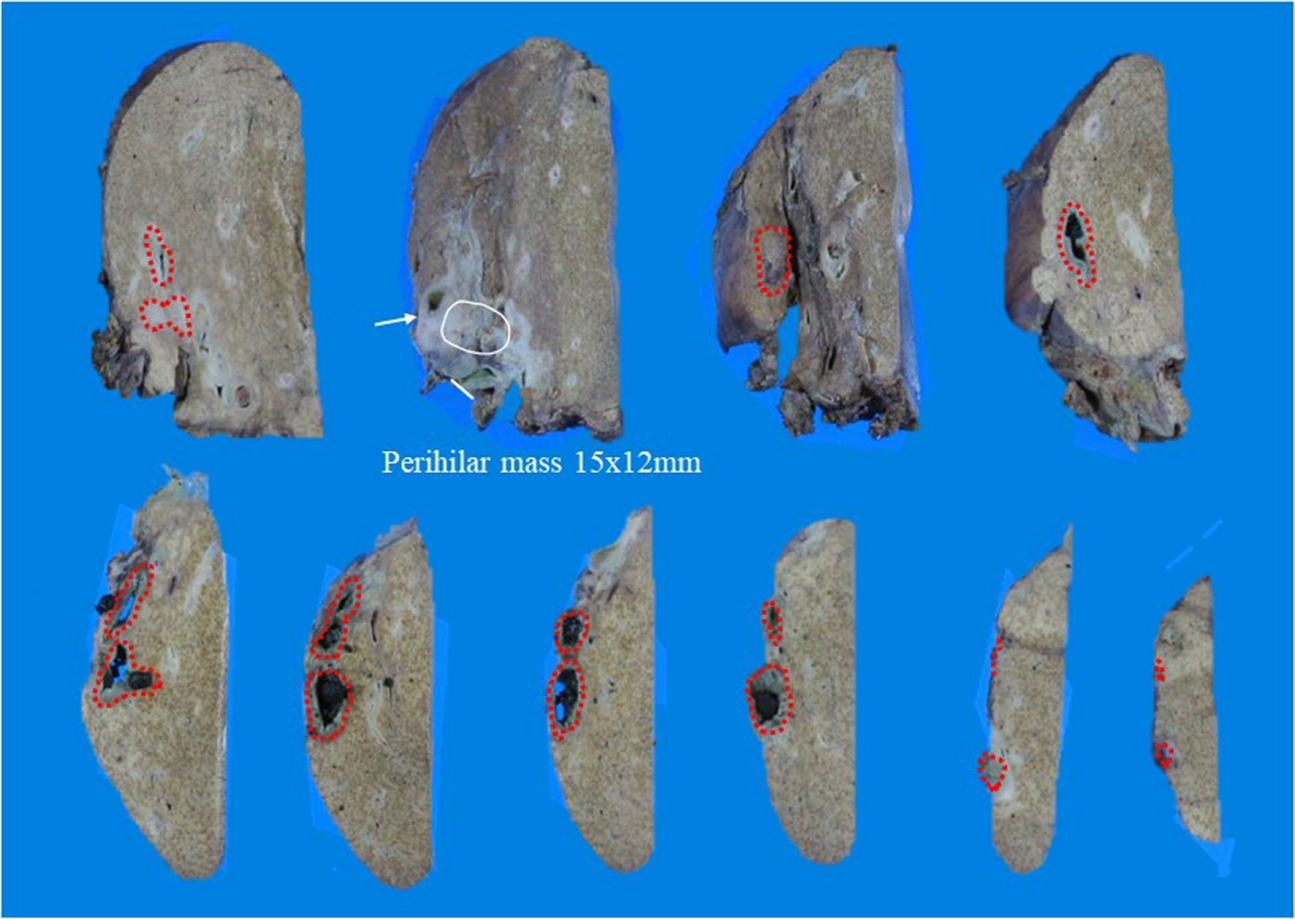
Macroscopic image of the cut surface of the resected specimen. A whitish solid tumor measuring 15 × 12 mm was observed adjacent to the dorsal side of the right hepatic hilum, involving the posterior glissonean pedicle (white circle). The posterior glissonean pedicle showed wall thickening (arrow), and this was associated with diffuse dilatation of the segment 6 bile ducts (dotted red circle). No gross intraductal tumor was noted in the dilated bile ducts. The white bar indicates the transection line of the right hepatic duct. The margin of the bile duct was approximately 1 cm from the mass

**Fig. 6:**
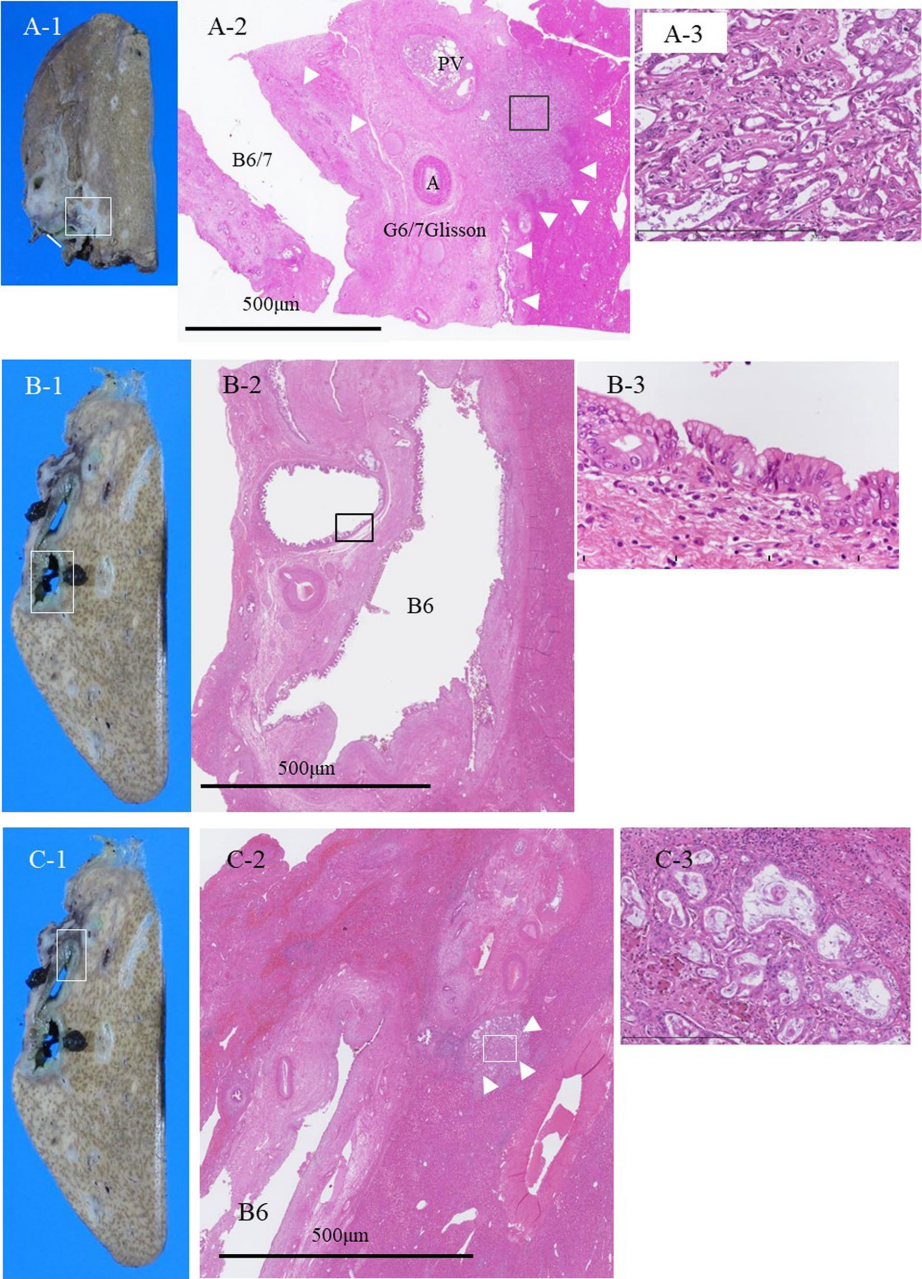
Macroscopic and histopathologic image of the specimen. **6A-1** Sagittal slice of the specimen at the level of the right hepatic hilum. A whitish solid and poor circumscribed tumor involved the right hepatic hilum (arrow) and extended along the posterior glissonean pedicle (arrowheads). The white line indicates the surgical cut margin of the bile duct. **6A-2** Tumor involved the posterior glissonean pedicle and surrounding hepatic parenchyma (arrowheads). H.E. staining. Scale bar = 500 µm. **6A-3** Tumor was composed of moderately differentiated tubular adenocarcinoma with a desmoplastic reaction. Perineural and lymphovascular invasion were observed. H.E. staining. Magnification of × 400. **6B-1** Sagittal slice at the middle of the segment 6. Grossly, the segment 6 bile ducts were cystically dilated, and no gross intraductal tumor was identified. **6B-2** Micropapillary or sessile proliferations of columnar epithelial cells were diffusely observed. The length of intraepithelial extension was approximately 120 mm. H.E. staining. Scale bar = 5 µm. **6B-3** Neoplastic lesions exhibited marked nuclear atypia, loss of nuclear polarity, and presence of mitoses, leading to a diagnosis of high-grade BilIN. H.E. staining. Magnification of × 400. **6C-1** At the peripheral slice of the segment 6. Similarly, diffuse bile duct dilatation was observed at the peripheral side of segment 6. **6C-2** Minimally invasive foci of atypical epithelial cells were observed in the peripheral hepatic parenchyma of segment 6 (arrowheads). The maximum size of invasion was approximately 2.1 mm in diameter. H.E. staining. Scale bar = 5 µm. **6C-3** Moderately differentiated tubular adenocarcinomas with desmoplastic reactions were observed. H.E. staining. Magnification of × 400.

**Fig. 7:**
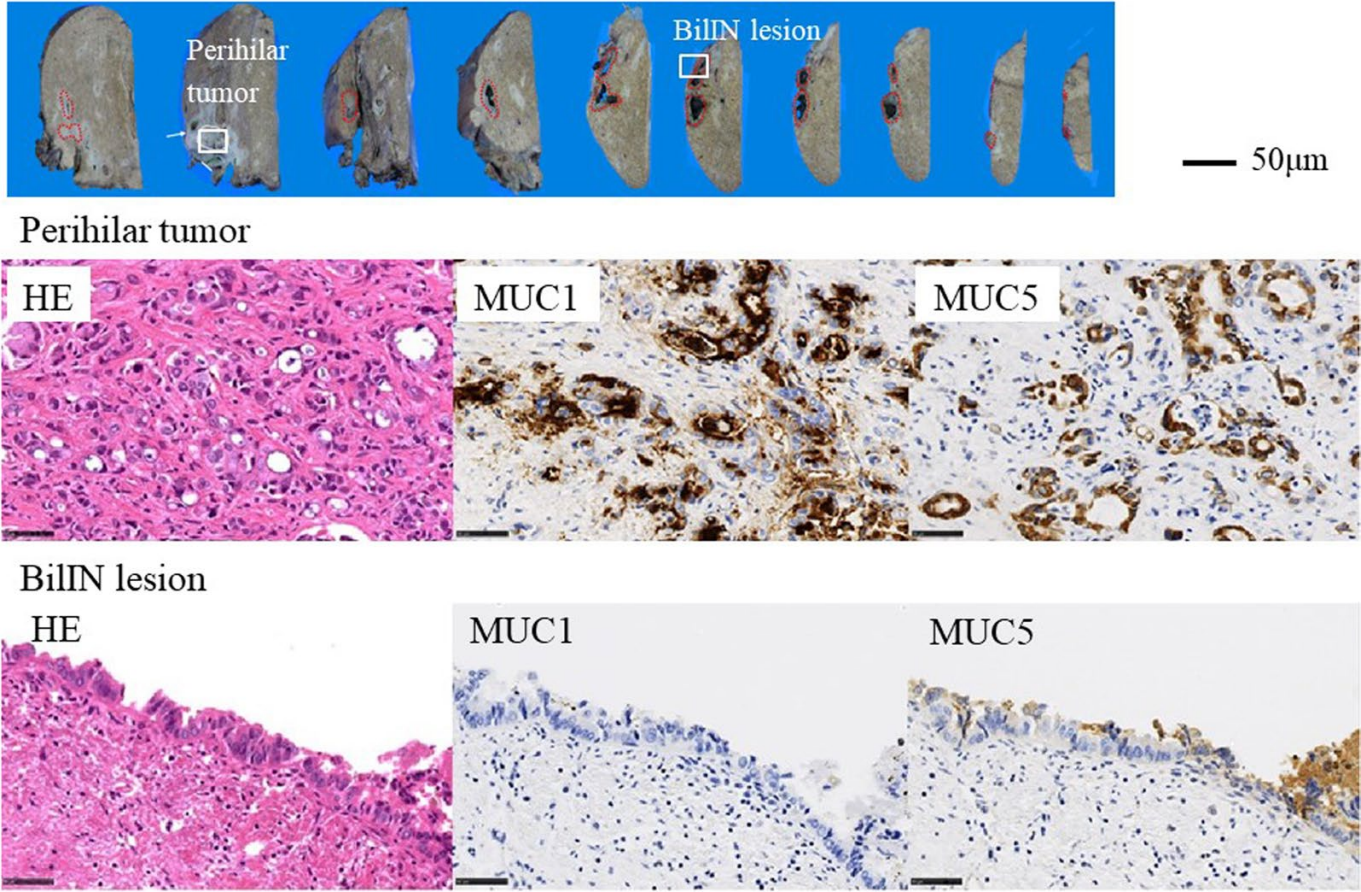
Immunohistochemical image of the specimen. The perihilar tumor exhibited strong positivity for MUC1 and MUC5AC. The BilIN lesions showed negativity for MUC1 and positivity for MUC5AC

**Fig. 8:**
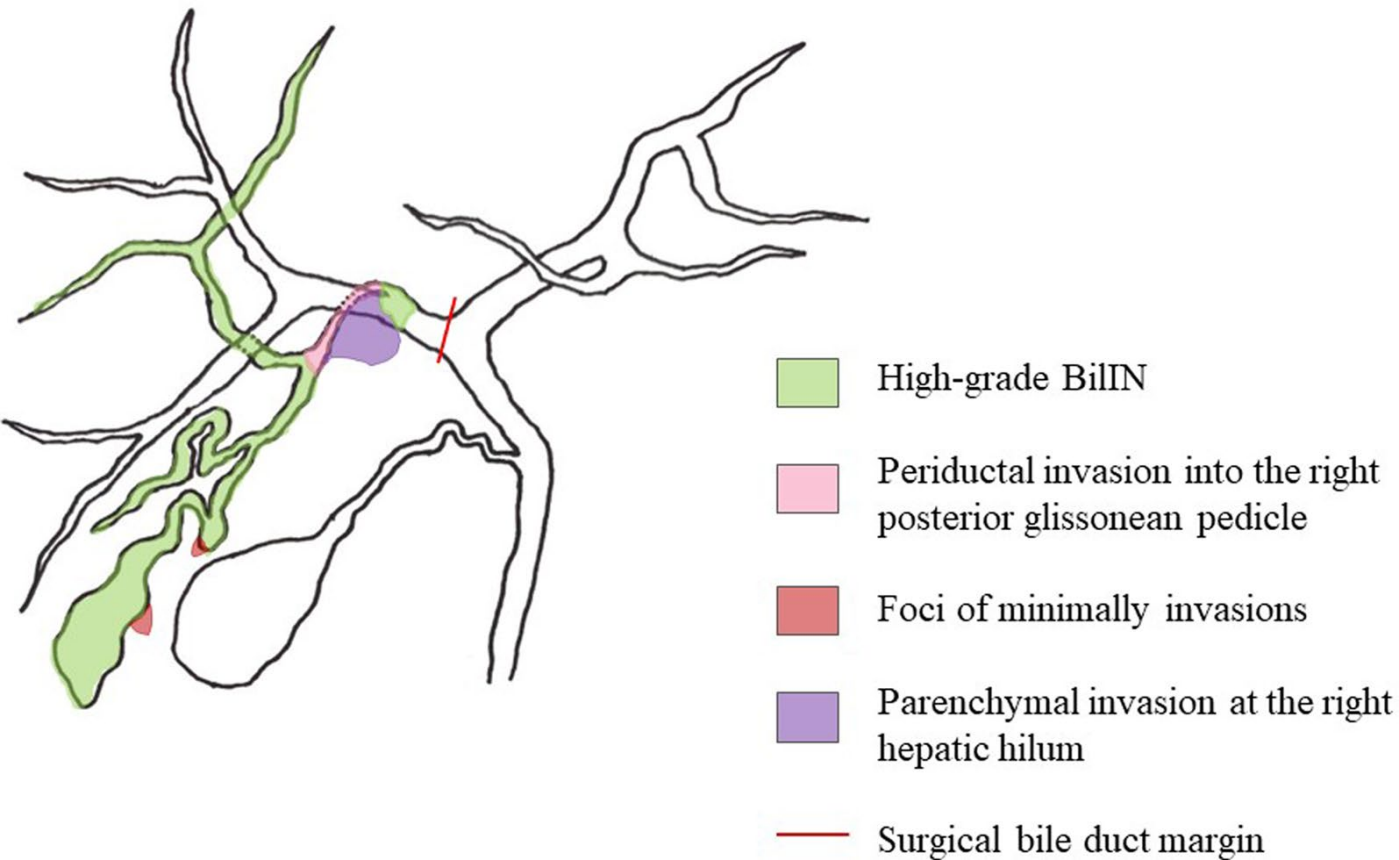
Schematic presentation of the distribution of the high-grade BilIN lesions, two foci of minimally invasions, and the perihilar intrahepatic cholangiocarcinoma

A 70-year-old female presented to our hospital with complains of appetite loss and epigastric discomfort. Physical examination revealed unremarkable findings. Laboratory examination of blood showed: carbohydrate antigen 19-9 (CA19-9) level, 1369.1U/mL (> 37 U/mL); DUPAN-2, 330 U/ml (> 150 U/mL); and Span-1 210 U/ml (> 30 U/mL). Liver function tests were within normal range. On computed tomography (CT), an enhancing tumor measuring 15 mm was observed, which extended to the right secondary biliary confluence (Fig. 1A). The tumor was also associated with wall thickening of the right posterior glissonean pedicle, in conjunction of the perihilar tumor and a cystic dilatation of the segment 6 bile duct (B6), measuring 7 mm in diameter (Fig. 1B). No findings suggestive of lymphadenopathy or distant metastasis were noted. Magnetic resonance cholangiopancreatography (MRCP) revealed stenosis at the bifurcation of the posterior glissonean pedicle and diffuse dilatation of the B6. On aT1-weighted contrast-enhanced image, a 15 mm enhancing mass was observed on the dorsal side of the right hepatic hilum, extending to the posterior glissonean pedicle (Fig. 2A). The tumor was associated with irregular wall thickening at bifurcation of the posterior glissonean pedicle (Fig. 2B). Fluoro-deoxy-glucose–positron emission tomography (FDG–PET)–CT scan revealed an abnormal accumulation of FDG in the mass with a maximal standardized uptake value (SUV) of 4.6. Endoscopic retrograde cholangiopancreatography (ERCP) demonstrated a stricture at the bifurcation of the segment 6 (B6) and 7 (B7) bile ducts (Fig. 3). Any intraductal neoplastic lesions were not identified. Mapping biopsies of the bile duct were not performed. Nonetheless, the diagnosis of adenocarcinoma was confirmed through bile duct cytology (Fig. 4). Based on these findings, the patient was diagnosed with a MF + PI type ICC involving the right posterior glissonean pedicle. The imaging studies, including cholangiography or MRCP, revealed no evidence of infiltrative changes such as sclerosis and narrowing in the root of right hepatic duct. Therefore, an R0 resection was anticipated without requiring additional resection of the extrahepatic bile duct. The patient underwent a right hepatectomy and lymph node dissection along with hepatoduodenal ligament, postpancreatic head and common hepatic artery. Macroscopically, a 15 × 12 mm whitish, poorly circumscribed solid tumor was observed adjacent to the dorsal side of the posterior glissonean pedicle (Fig. 5). Continuous with the perihilar tumor, the bile ducts at the intrahepatic secondary biliary confluence showed wall thickening with a whitish appearance. No gross intraductal tumor was observed in the dilated bile ducts. Histopathologically, the tumor was diagnosed as moderately differentiated adenocarcinomas with an irregular tubular pattern, exhibiting perineural and lymphovascular invasion (Fig. 6A). Diffuse micropapillary or sessile proliferations of columnar epithelial cells were observed in the posterior bile ducts. These lesions exhibited marked nuclear atypia, loss of nuclear polarity, and presence of mitoses, leading to a diagnosis of high-grade BilIN (Fig. 6B). Immunohistochemical results of the perihilar tumor showed MUC1 (2 +), MUC2 (-) and MUC5AC (3 +), and the BilIN lesions exhibited MUC1 (-). MUC2 (-), and MUC5AC (3 +) (Fig. 7). The length of intraepithelial extension was approximately 120 mm. The high-grade BilIN lesions demonstrated continuous extension, and a transition between the BilIN lesions and the perihilar tumor was observed. Furthermore, two foci of invasive atypical epithelial cells were noted in peripheral hepatic parenchyma. The maximum size of each observed cell clusters was approximately two millimeters in diameter (Fig. 6C). Small clusters of atypical epithelial cells were observed in one regional lymph node. No neoplastic lesions were identified at the bile duct margin, and a 10 mm resection margin was achieved. This suggests that the BilIN lesions originated from a large duct, extended in the bile duct and involved the posterior glissonean pedicle, leading to diagnosis as a MF + PI type ICC. A schematic representation of the distribution of the high-grade BilIN, two foci of minimally invasions in the peripheral hepatic parenchyma, and a perihilar mass lesion is depicted in Fig. 8. The pathological diagnosis was ICC pT4N1M0 Stage IVA according to the TNM classification system. Adjuvant chemotherapy was administered using gemcitabine and S-1 for 12 months. As of the 5 years following resection, no recurrence has been observed.

## Discussion

We present a rare case of ICC that is associated with extensive longitudinal intraductal extension of high-grade BilIN. This case is unique, because the epithelial tubular neoplasm, characterized by severe nuclear atypia, exhibited diffuse and continuous extension throughout the entire bile duct of the posterior segment. In addition, this lesion secondarily involved in the perihilar hepatic parenchyma. Progressive stages of this condition are also associated with two foci of minimally invasions in the peripheral hepatic parenchyma. In this case, the tumor exhibited perineural and lymphovascular invasions, and one regional lymph node metastasis was observed.

Anatomically, ICCs are categorized as small duct ICC or large duct ICC [4–6, 10]. Large duct ICC emerges from epithelial precursors, while no definite precursor lesion has been elucidated for small duct ICC. Morphologically, the main growth patterns of ICC have been categorized as MF, PI, or IG type according to the classification of the Liver Cancer Study Group of Japan [1]. The MF type is characterized by a defined mass within the liver parenchyma, and is the most common form of ICC, accounting for 78.6–90.0% of cases [10, 11]. IG type is the rarest type of ICC (approximately 6%) and typically presents as a papillary tumor or tumor thrombus within the dilated bile duct lumen [10]. Small duct ICCs are exclusively of the MF type. On the other hand, large duct ICC may often present as either the PI or IG type, but may also exhibit a MF pattern, either alone or in combination with other types as seen in this case. Adopted from pancreatic counterparts, precursors of bile duct cancer are divided into two main categories: BilIN and IPNB. The present case is characterized by a high-grade BilIN (carcinoma in situ) micropapillary or sessile neoplasm, with sever dysplastic epithelial cell growth that was diffusely observed in the posterior bile ducts. The present case can be classified as a MF + PI type ICC that accompanied by hepatic parenchymal invasion at the right hepatic hilum. In this case, in addition to the hepatic parenchymal invasion observed at the right hepatic hilum, two distinct clusters of invasive atypical epithelial cells were also observed in the peripheral hepatic parenchyma. Significant associations of morphologic subtypes and tumor spread patterns with patient prognosis have been reported [10, 12–14]. The 3-year disease-free survival of ICC in the case of MF, PI, and IG type ICC is 29%, 30%, and 61%, respectively [13]. The combination of PI type and extensive superficial spreading high-grade BilIN lesions has been rarely reported, and the prognosis of its mixed variants is not well-known.

BilIN has been reported as a preneoplastic condition that has the potential to progress to invasive cholangiocarcinoma [5–9]. Pathogenesis of BilIN involves prolonged inflammation followed by sequential accumulation of genetic and epigenetic alterations and malignant transformation of the biliary epithelium [8, 9]. Most data have been collected in association with other conditions, such as cholelithiasis, hepatolithiasis, primary PSC, cirrhosis, and so on [7–9]. In regions where cholelithiasis is endemic, the prevalence of low-grade and high-grade BilINs in cholecystectomy specimens is reported to be about 15% and up to 3.5%, respectively, compared to < 5% and < 0.1% as reported in North America [15]. While high-grade BilIN lesions are commonly observed in the vicinity of cholangiocarcinoma, the epidemiology of BilIN lesions in the bile ducts has not been extensively reported. In this case, no inflammatory conditions associated with reported carcinogenic risks were observed. The detailed examination of the extent and degree of BilIN lesions supports the understanding of the underlying disease progression and aids in the specific classification of PI type ICC.

Immunohistochemical results of the BilIN lesions showed negative expression of MUC1 and MUC2, but positive expression of MUC5AC ( +). In contrast, the perihilar tumor exhibited positive for MUC1 and MUC5AC, but negative for MUC2. Zen Y et al. reported different expression patterns of MUCs and cytokeratins in neoplastic biliary epithelia of BilIN and IPNB during progression to ICC [16]. Frequent expression of MUC1 has been associated with the development of tubular adenocarcinoma, while MUC2 expression indicative of the presence of the intestinal metaplasia, which is frequently observed in IPNB lineage. Carcinogenesis via BilIN, predominantly associated with tubular adenocarcinoma, is characterized by a negative expression of MUC2 and an increase in the expression of MUC1, indicating the MUC1-positive pathway. On the other hand, the IPNB pathway, which frequently progresses to colloid adenocarcinoma, is characterized by a positive expression of MUC2, indicating the MUC2-positive pathways [17]. In cases of BilIN, MUC5AC expression tends to become more extensive with increasing degrees of BilIN, particularly in patients with high-grade BilIN, as observed in the present case. Indeed, the identification of these pathways through immunophenotyping of MUCs and cytokeratins can offer valuable insights to clinicians in terms of the progression and prognosis of biliary neoplasms [16]. A higher expression of MUC1 and MUC5AC in tumors is associated with increased cell proliferations and is closely related to poor prognosis [16, 17]. The patient achieved a 5-year disease free survival (DFS). While the clinical benefits of adjuvant chemotherapy for ICC are not yet well-defined, there has been growing attention towards utilizing adjuvant chemotherapy to enhance surgical prognosis [18, 19]. Potential survival benefits of adjuvant chemotherapy might be associated with tumor subgroups, as observed in the current case, such as the presence of lymph node metastasis.

Lymph node metastasis (LNM) has been one of the strongest predictors of poor outcome among patients undergoing curative-intent resection for cholangiocarcinoma [20–22]. Zhang XF et al. reported that patients with at least one LNM had a worse overall survival (OS) compared to patients without LNM who underwent curative-intent resection for ICC (median OS, LNM 18.0 vs no nodal disease 45.0 months, P < 0.001) [21]. LNM is common, as it was reported in 30–40% of patients overall who underwent resection and lymph node dissection (LND) [20–22]. LNM is more frequently observed in PI type ICC, with a prevalence over 60%, which is higher than the rates observed in other types [13, 20]. PI type ICC frequently extends towards the hepatic hilum, invading the portal connective tissue and the hepatic parenchyma. This invasive behavior can lead to perineural and lymphatic invasion, as well as lymph node metastasis. Although LNM is a significant predictor of poor prognosis, the evidence of therapeutic benefit from LND is insufficient and no consensus has been reached regarding whether lymphadenectomy should be routinely performed. Umeda Y et al. reported that hilar lesions exhibited the highest rate of LNM at 44% (57 out of 130). They discussed the therapeutic implications of LND in cases of hilar lesions and suggested extending LND to include postpancreatic head or common hepatic artery nodes [23].

Surgical margins should also be carefully evaluated and taken into consideration during clinical procedures. In a recent meta-analysis investigating the effect of surgical margin width on OS in patients with ICC, it was found that negative surgical margins are associated with improved OS and DFS following surgical resection [22]. Specifically, patients with ICC and R0 margins ≥ 10 mm demonstrate a greater survival benefit compared to those with < 10 mm [22]. In the present case, despite the mass lesion appearing to be located close to the bifurcation of right hepatic glissonean pedicle, the preoperative imaging diagnosis, including cholangiography and MRCP, indicated the potential for achieving a negative bile duct transection margin. The intraoperative pathological examination further confirmed the absence of infiltrative lesions or high-grade dysplastic lesions in the examined bile duct. Finally, a 10 mm resection margin was achieved in the permanent pathological specimen. Meticulous assessment of surgical margins is crucial to ensure complete removal of the tumor and achieve negative margins. Distinguishing BilIN from reactive epithelial atypia can be challenging, particularly in cases with concurrent active inflammation, ulceration, or after stenting [22]. Therefore, in cases where tumor cells are suspected or identified at the surgical margin, a planned combined resection or additional resection of the extrahepatic bile duct should be considered.

## Conclusion

We present a case of ICC involving the right hepatic hilum, accompanied by extensive longitudinal high-grade BilIN lesions and multifocal microscopic invasions in peripheral hepatic parenchyma. The presence of biliary neoplasia with such extensive intraductal extension, in conjunction with ICC, should be considered as a variant of BilIN.

## Data Availability

Not applicable as this is a case report; all data presented were extracted from the patient’s hospital chart.

## Abbreviations

ICC: Intrahepatic cholangiocarcinoma
BilIN: Biliary intraepithelial neoplasia
IPNB: Intraductal papillary neoplasm of the bile duct
PSC: Primary sclerosing cholangitis
MF: Mass forming
PI: Periductal infiltrative
IG: Intraductal growth
CA19-9: Carbohydrate antigen 19-9
CT: Computed tomography
MRCP: Magnetic resonance cholangiopancreatography
FDG–PET: Fluoro-deoxy-glucose–positron emission tomography
SUV: Standardized uptake value
ERCP: Endoscopic retrograde cholangiopancreatography
DFS: Disease free survival
LNM: Lymph node metastasis
OS: Overall survival
LND: Lymph node dissection
